# Neuromelanin activates proinflammatory microglia through a caspase-8-dependent mechanism

**DOI:** 10.1186/s12974-014-0228-x

**Published:** 2015-01-14

**Authors:** Nikenza Viceconte, Miguel A Burguillos, Antonio J Herrera, Rocío M De Pablos, Bertrand Joseph, José L Venero

**Affiliations:** Department of Biochemistry and Molecular Biology, Faculty of Pharmacy, University of Sevilla, 41012 Sevilla, Spain; Instituto de Biomedicina de Sevilla, Hospital Universitario Virgen del Rocío/CSIC/Universidad de Sevilla, 41013 Sevilla, Spain; Present address: Department of Biosciences and Nutrition, Karolinska Institutet, 17177 Stockholm, Sweden; Centre for Neuroscience and Trauma, Blizard Institute, Queen Mary University of London, E1 2AT London, United Kingdom; Department of Oncology-Pathology, Karolinska Institutet, Cancer Centrum Karolinska, 17176 Stockholm, Sweden

**Keywords:** caspase-3, caspase-8, cytokines, microglia, neuroinflammation, Parkinson’s disease

## Abstract

**Background:**

We have uncovered a caspase-dependent (caspase-8/caspase-3/7) signaling governing microglia activation and associated neurotoxicity. Importantly, a profuse non-nuclear activation of cleaved caspases 8 and 3 was found in reactive microglia in the ventral mesencephalon from subjects with Parkinson’s disease, thus supporting the existence of endogenous factors activating microglia through a caspase-dependent mechanism. One obvious candidate is neuromelanin, which is an efficient proinflammogen *in vivo* and *in vitro* and has been shown to have a role in the pathogenesis of Parkinson’s disease. Consequently, the goal of this study is to test whether synthetic neuromelanin activates microglia in a caspase-dependent manner.

**Results:**

We found an *in-vivo* upregulation of CD16/32 (M1 marker) in Iba1-immunolabeled microglia in the ventral mesencephalon after neuromelanin injection. *In vitro* experiments using BV2 cells, a microglia-derived cell line, demonstrated that synthetic neuromelanin induced a significant chemotactic response to BV2 microglial cells, along with typical morphological features of microglia activation, increased oxidative stress and induction of pattern-recognition receptors including Toll-like receptor 2, NOD2, and CD14. Analysis of IETDase (caspase-8) and DEVDase (caspase-3/7) activities in BV2 cells demonstrated a modest but significant increase of both activities in response to neuromelanin treatment, in the absence of cell death.

**Conclusions:**

Caspase-8 inhibition prevented typical features of microglia activation, including morphological changes, a high rate of oxidative stress and expression of key proinflammatory cytokines and iNOS.

## Background

Caspases are a family of cysteinyl aspartate-specific proteases that mediate proteolytic events indispensable for the transduction of signaling pathway controlling biological phenomena, including apoptosis (programmed cell death), necrosis, proliferation, differentiation, and inflammation [1]. Activation of apoptotic caspases has been long interpreted as a commitment to cell death [2]. However, recent reports have demonstrated an increased number of non-apoptotic roles for these caspases [3–6]. We recently described a novel and unexpected role for caspase-8 -3 and -7 in the activation of microglia and associated neurotoxicity, a finding that could be relevant in the onset of chronic neurodegenerative diseases [7]. We found that activation of microglial cells with different proinflammogens, including lipopolysaccharide (a toll-like receptor (TLR) 4 agonist), lipoteichoic acid (a TLR2 agonist), PamC3sk4 (a TLR 1/2 agonist) and IFNγ lead to modest but yet significant caspase-3/7 activities in the absence of cell death. Caspase-8 was found to act as an upstream caspase in this signaling pathway, since chemical inhibition or gene knockdown using siRNAs of caspase-8 prevented caspase-3/7 activation and the subsequent appearance of typical molecular features of microglia activation [7]. We also analyzed post-mortem ventral mesencephalon from patients who had been diagnosed with Parkinson’s disease and found significant cytoplasmic expression for active caspase-3 and caspase-8 in CD68-immunoreactive microglia [7]. This observation is suggestive of the existence of endogenous factors capable of activating the microglia through a caspase-dependent mechanism.

Parkinson’s disease affects approximately 1 to 3% of the population, and is characterized by a slow and progressive degeneration of dopaminergic neurons in the substantia nigra [8]. Accumulating evidence suggests that inflammation may play a central role in the cell loss seen in Parkinson’s disease [9,10]. Epidemiological studies have demonstrated convincingly that the incidence of idiopathic Parkinson’s disease is lower with chronic use of anti-inflammatory drugs [11–13]. Evidence of extensive inflammatory response in Parkinson’s disease is well documented [14]. A number of studies reveal that damaged dopaminergic neurons release several factors that seem to activate microglia, including neuromelanin, α-synuclein, and μ-calpain [15,16]. Neuromelanin itself may induce a neurodegenerative process via an inflammatory mechanism, as extracellular neuromelanin has the ability to activate the central nervous system immune cells, microglia [17–19]. Released extracellular neuromelanin has been observed close to activated microglia cells in brains from patients with Parkinson’s disease as well as 1-methyl-4-phenyl-1,2,3,6-tetrahydropyridine (MPTP)-induced parkinsonism [20,21]. The immunological response of neuromelanin could damage other neurons and lead to a vicious cycle of neuroinflammation and neurodegeneration [17,22]. Consequently, we tested the ability of synthetic neuromelanin to activate BV2 microglia cells through a caspase-8-dependent mechanism. We found that caspase-8 is involved in the neuromelanin-induced activation of BV2 microglial cells.

## Methods

### Preparation of synthetic neuromelanin

Dopamine (1.5 g) and cysteine (232 mg) were dissolved in 1,000 ml of 50 mM phosphate buffer (pH 7.4). The molar ratio of dopamine to cysteine in this solution is 6:1. The solution of dopamine and cysteine was incubated at 37°C with free air access and vigorous stirring for 3 days. The resulting black pigment was collected after centrifugation at 10,000 rpm for 10 min, and washed with 1% acetic acid and twice with distilled water. To remove low molecular weight substances, the resulting synthetic neuromelanin was dialyzed in tubing with a molecular weight cut off of 12,000 to 14,000 (Sigma-Aldrich, San Luis, MO, USA), which had previously been treated with EDTA. Finally, the neuromelanin was washed twice with distilled water and kept at 4°C until use.

### Animals and surgery

C57BL/6 mice were obtained from Seville University (Spain) and had free access to food and water. Experiments were performed in accordance with the Guidelines of the European Union Directive 2010/63/EU, following Spanish regulations for the use of laboratory animals, and were approved by the Scientific Committee of the University of Seville.

Intranigral neuromelanin injections (4 μg in 1 μl sterile saline) were made 1.2 mm posterior, 1.2 mm lateral, and 5.0 mm ventral to the lambda. Two days later, mice were transcardially perfused under deep anesthesia with 4% paraformaldehyde and PBS, pH 7.4. Brains were removed, cryoprotected in sucrose and frozen in isopentane at −15°C and serial coronal sections (25 μm thick) covering the substantia nigra were cut with a cryostat, mounted on gelatin-coated slides, and further processed for immunohistochemical analysis of the microglia.

### Cell culture

BV2 cells were cultured in DMEM (Invitrogen, Carlsbad, CA, USA) supplemented with 10% heat-inactivated FBS (Sigma-Aldrich, San Luis, MO, USA), streptomycin (100 mg/ml), and penicillin (100 IU/ml) under 100% humidity and 5% CO_2_. Experiments were performed in reduced 5% FBS media.

### Cell survival assay

To measure cell viability, 3-(4,5-dimethylthiazol-2-yl)-2,5-diphenyl tetrazolium (MTT) (Sigma-Aldrich, San Luis, MO, USA) assays were conducted. Briefly, BV2 cells were plated at a density of 1 × 10^4^ cells/ml in 96-well plates. After incubation for 24 h, the cells were washed with fresh medium supplemented with 5% FBS and treated with different concentrations of synthetic neuromelanin for 12, 24, and 36 h in a humidified incubator. Then 10 μl of MTT solution (2 mg/ml) were added to each well and cultured for 3 h. After that, the supernatant was removed and the formazan product obtained was dissolved in 100 μl dimethylsulfoxide (Sigma-Aldrich, San Luis, MO, USA). The absorbance was measured at 570 nm using a microplate reader. Cell viability in the treated cells is expressed as a percentage of the viability of untreated cells.

### Assay for total nitrite

The cells were plated on 24-well dishes at a density of 10^5^ cells per well and after 24 h were treated with 1 μg of neuromelanin for 24 h. The total nitrite content in the supernatant was measured by incubating supernatants with 20 μl of 2,3-diaminonaphtalene solution in hydrochloric acid and incubating for 10 min at room temperature, protected from light. The reaction was stopped by adding 100 μl NaOH, and the total amount of nitrite was measured spectrophotometrically using a microplate reader at an excitation wavelength of 363 nm and an emission wavelength of 426 nm. The nitrite concentration was calculated using standards prepared from nitrite standard solution.

### Chemotaxis assay

Migration properties of BV2 were evaluated in the presence and absence of synthetic neuromelanin. The test was performed using the Chemicon QCM™ Chemotaxis 5 μm 24-well migration assay (Catalog number ECM507, CHEMICON® International, Millipore), which is based on the Boyden chamber principle.

The BV2 cells were resuspended in serum-free DMEM and plated at a concentration of 5 × 10^5^ cells into the upper compartment of a 24-well transwell plate (5.0 nm pore size), while to the lower compartment was added serum-free DMEM plus 0.5 and 1 μg of neuromelanin and incubated for 21 h at 37°C and 5% CO_2_. Cells in the lower chamber were lysed and cell numbers were determined using CyQUANT® GR dye. The fluorescence signal was read with a plate reader using a 480/520 nm filter set. 10% FBS was used as a positive control in the assay because it is a well-established chemotactic substance for macrophages and monocytes.

### Determination of intracellular reactive oxygen species content

The intracellular reactive oxygen species (ROS) content, induced by neuromelanin treatment, was determined in live cells using the fuorogenic ROS sensor CellROX™ Deep Red Reagent (Invitrogen) at a final concentration of 5 μM. After 30 min of incubation at 37°C, the medium was removed, the cells were washed three times with PBS, and the resulting fluorescence was analyzed by confocal microscopy (Zeiss LSM 7 Duo).

### Determination of apoptotic cell death by propidium iodide and annexin V-FITC staining

BV2 cells were treated with 1 μg/ml of neuromelanin for 24 h at 37°C. After treatment, cells were washed twice with cold PBS and resuspended in Binding Buffer (BD Biosciences, Franklin Lakes, NJ, USA) at a concentration of 5 × 10^5^ cells/ml in 300 μl. Then BV2 cells were incubated with 5 μl of V500 annexin V (BD Biosciences) for 30 min and with 1 μg/ml of propidium iodide for 1 min at room temperature in the dark. The reported graph is representative example of three experiments, which all gave very similar results.

### Caspase 3 and 8 activity assay

Caspase activities were measured using the Caspase-Glo® kits from Promega (Madison, WI, USA) following the manufacturer protocol. For that, BV2 cells were seeded at a density of 5 × 10^5^/well for 24 h and then treated with 1 μg/ml of synthetic neuromelanin for 24 h and the caspase activities measured while the comparing counterpart were first pre-treated with Z-Ile-Glu(OMe)-Thr-Asp(Ome)-fluoromethylketone (IETD)-fmk for 30 min and then exposed to neuromelanin.

### Real-time PCR

The expression levels of selected gene encoding for membrane surface receptors (TLR4, TLR2, nucleotide-binding oligomerization domain-containing protein (NOD)1, NOD2, receptor for advanced glycation end products (RAGE), and CD14) and proinflammatory cytokines (such as IL-6, iNOS, IL-1β, and TNF-α) were quantified using real-time PCR. The BV2 cells were seeded at a density of 5 × 10^5^ per well for 24 h and then treated with 1 μg/ml of neuromelanin for 6 h and 24 h. Total RNA was isolated from cells using Rneasy® kit (Qiagen, Hilden, Germany). cDNA was synthesized from 1 μg of total RNA using a QuantiTect® reverse transcription kit (Qiagen) in 20 μl reaction volume, as described by the manufacturer. Real-time PCR was performed using a SensiFAST™ SYBR No-rox kit (Bioline, London, UK), 0.4 μM primers and 1 μl cDNA. Controls were carried out without cDNA. Amplification was run in a Mastercycler® ep realplex (Eppendorf, Hamburg, Germany) thermal cycler at 94°C for 2 min followed by 35 cycles of 94°C for 30 s, 55 to 60°C for 45 s, and 72°C for 45 s, followed by a final elongation step at 72°C for 7 min. Following amplification, a melting curve analysis was performed by heating the reactions from 65 to 95°C in 1°C intervals while monitoring fluorescence. Analysis confirmed a single PCR product at the predicted melting temperature. *GAPDH* served as the reference gene and was used for sample normalization. The primer sequences used for amplifications are listed in Table 1.

**Table 1:** **Primers for RT-PCR**

**Target mRNA**	**Forward (F) and reverse (R) primers**
TLR4	F: 5-GGA CTC TGA TCA TGG CAC TG-3
	R: 5-CTG ATC CAT GCA TTG GTA GGT-3
TLR2	F: 5-GGG GCT TCA CTT CTC TGC TT-3
	R: 5-AGC ATC CTC TGA GAT TTG ACG-3
CD14	F: 5-AAA GAA ACT GAA GCC TTT CTC G-3
NOD1	R: 5-AGC ACC AAG CCA AGC ACA C-3
NOD2	F: 5-AGT GAG GGC GGG AAG TGT-3
	R: 5-CCA ATC TGA TTA CCC CAC ATC-3
	F: 5-CCC TAG CAC TGA TGC TGG A-3
	R: 5-CCC CTT CGT CAC AGA TAT GG-3
RAGE	F: 5-GTG TCG GGC AAC TAA CAG G-3
	R: 5-CTG GCT TCC CAG GAA TCT G-3
IL-6	F: 5-TTC TTG GGA CTG ATG CTG-3
	R: 5-CTG GCT TTG TCT TTC TTG TT-3
IL-1β	F: 5-TTG ACG GAC CCC AAA AGA TG-3
	R: 5-AGA AGG TGC TCA TGT CCT CA-3
iNOS	F: 5-CTT TGC CAC GGA CGA GAC-3
	R: 5-TCA TTG TAC TCT GAG GGC TGA C-3
TNF-α	F: 5-AGC CCA CGT AGC AAA CCA CC-3
	R: 5-AAC ACC CAT TCC CTT CAC AGA GC-3
GPDH	F: 5-AAC GAC CCC TTC ATT GAC C-3
	R: 5-TCA GAT GCC TGC TTC ACC-3

The relative differences in expression between treated and untreated cells were determined using cycle time (Ct) values first normalized with *GAPDH* of the same sample and then, the relative differences between control and treated cells were calculated and expressed as a relative increases setting control as 100%.

Data were collected and analyzed using the provided application software. All of the PCR experiments were performed in triplicate to verify the results.

### Flow cytometry analysis

The fluorescent signals were analyzed using an LSRFortessa cytometer (BD Biosciences, Franklin Lakes, NJ, USA) equipped with a 50 mW 488 nm laser, a 40 mW 640 nm red laser, and a 50 mW 405 nm violet laser. The fluorescence emissions were detected through a 530/30 nm band-pass filter for fluorescein isothiocyanate conjugated antibody (FITC, FL1), a 610/20 nm band-pass filter for propidium iodide (FL3) and a 670/14 nm band-pass filter for allophycocyanin (FL12). At least 10,000 events per sample were acquired in log mode. Percentages of cells were calculated using fluorescence-activated cell sorting and Diva Software (BD Biosciences, Franklin Lakes, NJ, USA).

### Fluorescence immunohistochemistry

Thaw-mounted 20-μm coronal sections were cut on a cryostat at −15°C and mounted in gelatin-coated slides. For double-labeling of Iba-1 with CD16/CD32 (CD16/32), sections were blocked with PBS containing 1% normal goat serum and chicken serum (Vector Laboratories, Burlingame, CA, USA) for 1 h. The slides were washed three times in PBS and then incubated overnight at 4°C with either rabbit-derived anti-Iba-1 (1:300; Wako) or rat-derived anti-CD16/32 (1:500; BD Biosciences) diluted in PBS containing 1% normal goat or chicken serum and 0.25% Triton X-100. Sections were incubated with goat anti-rabbit secondary antibody conjugated to fluorescein (1:200, for Iba1; Vector) and chicken anti-rat secondary antibody conjugated to Alexa Fluor® 594 (1: 200, for CD16/32; Invitrogen) for 1 h at 22 ± 1°C in the dark. This addition was preceded by three 10-min rinses in PBS. As a control, another set of sections was incubated with only the Iba-1 antibody and then visualized using both filters. No signal was detected when Iba-1 alone with fluorescein filter was used (photomicrograph not shown). The same was true with CD16/32 when Alexa Fluor® 594 filter was used. Fluorescence images were acquired using a confocal laser scanning microscope (Zeiss LSM 7 DUO) and processed using its associated software package (ZEN 2010).

### Statistical analysis

All data were collected from three or more independent experiments and values were expressed as means ± standard deviation. Statistical analysis was performed using Student’s *t* test and one-way analysis of variance (ANOVA). Values of *P* less than 0.05 were considered statistically significant.

## Results

### Effect of intranigral injection of neuromelanin on microglia population

**Figure 1:**
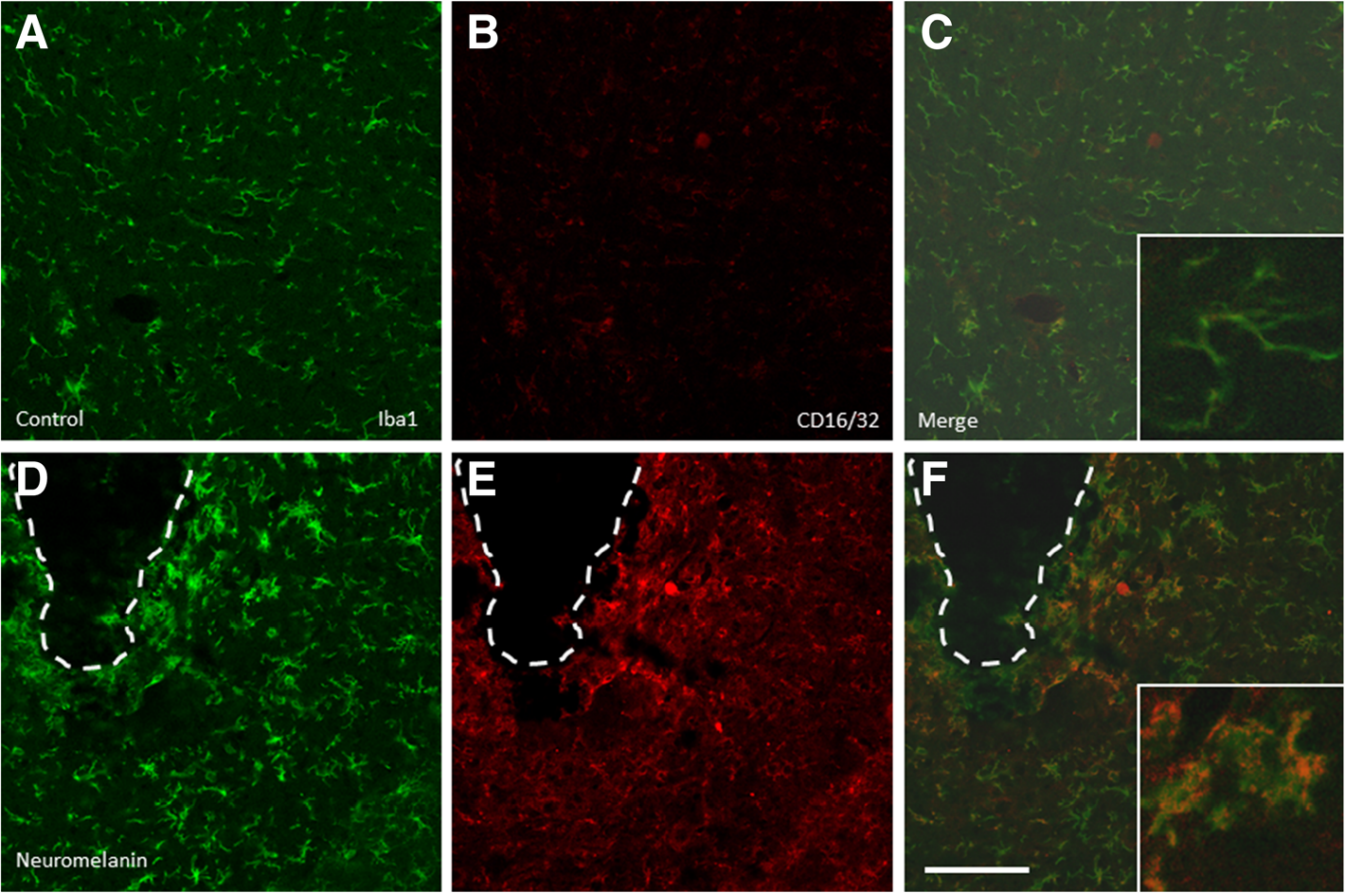
**Effect of** ***in-vivo*** **intranigral injection of neuromelanin on microglia population.** Microglia population was evaluated in terms of Iba1 **(A,D)** and CD16/32 **(B,E)** in sham **(A-C)** and neuromelanin-injected animals **(D-F)**. Inserts in C and F are high-magnification photographs of each condition. Note the typical morphological features of activated microglia in response to neuromelanin injections in terms of Iba1 **(D)** and upregulation of CD16/32 **(E)**. The dashed line in **D-F** shows the presence of insoluble neuromelanin within the ventral mesencephalon. Scale bar, 100 μm.

We first tested the effect of intranigral neuromelanin injections on microglia population in terms of Iba1 (general microglia marker) and CD16/32 (specific M1 proinflammatory marker). In sham-injected animals, the microglia were mostly quiescent, characterized by small cell bodies, fine cytoplasmic ramifications, and low to moderate Iba1 expression (Figure 1). CD16/32 expression was very low, supporting the quiescent nature of microglia in sham-injected animals. In contrast, neuromelanin injection strongly activated microglia; that is, cells showing thickening of processes and increased cell body size and Iba1 expression (Figure 1). Moreover, there was a strong induction of CD16/32 expression within Iba1-expressing microglial cells, thus supporting a proinflammatory phenotype (Figure 1).

### Effects of synthetic neuromelanin on BV2 cells viability

**Figure 2:**
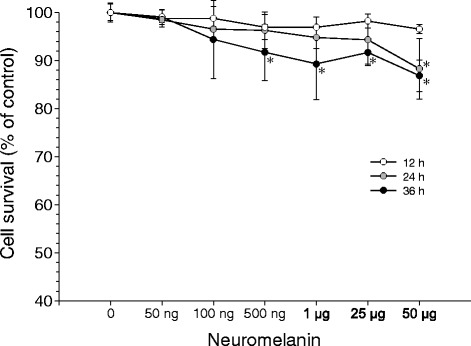
**Effects of synthetic neuromelanin on BV2 cell viability.** Results are mean ± standard deviation of three independent experiments for each concentration and time point assayed, and are expressed as a percentage of control values (vehicle instead of synthetic neuromelanin for each time point). Statistical analysis: one-way ANOVA followed by the least significant difference post-hoc test for multiple comparisons. α = 0.05 was used. *F* (20,62) = 3.38; *, compared with control values, *P* = 0.0004.

**Figure 3:**
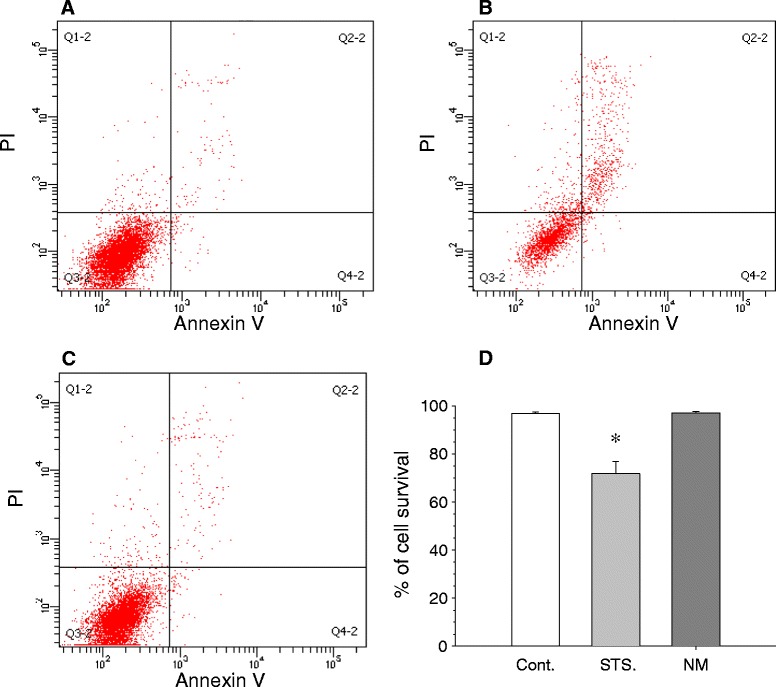
**Flow-cytometric analysis of BV2 cell viability.** Cells were analyzed using propidium iodide and annexin V. **(A)** Control cells. **(B)** Staurosporine (1 μM for 12 h, as death control). **(C)** BV2 cells + 1 μg/ml of neuromelanin for 24 h. **(D)** Quantification of cell survival. Results are mean ± standard deviation of three independent experiments, and are expressed as percentage of control values. Statistical analysis was made using Student’s *t* test. *, *P* < 0.01 compared with control values. Cont., control; NM, neuromelanin; PI, propidium iodide; STS, staurosporine.

A dose-response study of cell viability (MTT assay) after neuromelanin treatment was performed at three time points (12, 24, and 36 h) to establish the optimal concentration of synthetic neuromelanin to be used in the following studies on microglia activation. Neuromelanin was used in a range from 50 ng to 50 μg. Even the highest dose tested (50 μg) failed to decrease cell survival at 12 h (Figure 2), but produced a significant decrease at 24 h (86.3% of control value). At 36 h, a significant decrease in viability was found from 500 ng onward. We selected a dose of 1 μg/ml of neuromelanin, since it was able to activate microglia cells at short times (12 to 24 h) without inducing cell death, as confirmed by flow cytometry analysis with annexin V and propidium iodide (Figure 3).

### Neuromelanin stimulates chemotaxis of BV2 cells

**Figure 4:**
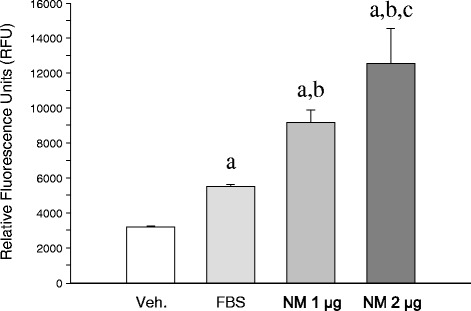
**Chemotaxis of BV2 cells induced by synthetic neuromelanin.** Results are mean ± standard deviation of three independent experiments, and are expressed as relative fluorescent units. Statistical analysis: one-way ANOVA followed by the least significant difference post-hoc test for multiple comparisons. *α* = 0.05 was used. *F* (3,11) = 45.35, *P* = 0.0000. All treatments are statistically different from each other.

Microglia are migratory cells that accumulate *in vivo* at sites of tissue injury. This property is important for many pathophysiological processes, including immune defense and wound healing. The effect of neuromelanin on microglial cell migration was measured by a cell migration assay based on the Boyden chamber principle. The migration of BV2 microglial cells across a membrane, measured after an incubation of 21 h, increased with the presence of neuromelanin in the medium in a dose-depending manner, nearly 2- and 3-fold for 1 and 2 μg of synthetic neuromelanin, respectively, compared with vehicle (Figure 4).

### Activation of nitric oxide production by neuromelanin

**Figure 5:**
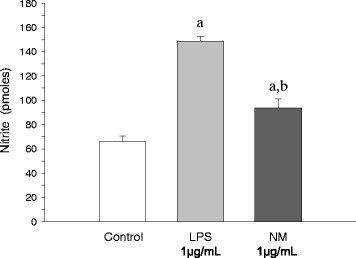
**Activation of nitric oxide production by neuromelanin.** Results are mean ± standard deviation of three independent experiments, and are expressed as pmol of nitrite. Statistical analysis: one-way ANOVA followed by the least significant difference post-hoc test for multiple comparisons. *α* = 0.05 was used. *F* (2,8) = 168.02, *P* < 0.01. All treatments are statistically different from each other.

Features of microglial activation are the production of nitric oxide, ROS, and proinflammatory factors. Consequently, microglia activation was first evaluated in terms of released nitrite upon neuromelanin treatment. Total nitrite levels were measured in the supernatant obtained from BV2 cells incubated with 1 μg/ml of synthetic neuromelanin. A significant increase of 42.4% of nitrite production was detected after 24 h of neuromelanin exposure, as compared with control cells (*P* < 0.01, Figure 5). For comparative purposes, we also analyzed nitrite levels after treating BV2 cells for 24 h with 1 μg/ml lipopolysaccharide (125.0% increase, *P* < 0.01 compared with control levels).

### Expression of pattern-recognition receptors in response to synthetic neuromelanin

Activation of microglial cells largely relies upon binding to and stimulation of pattern-recognition receptors, thus leading to a high transcriptional response, including the receptors themselves. We used RT-PCR to quantify the mRNA levels encoding for key surface receptors, including TLR2 and TLR4, CD14, RAGE, and NOD1 and NOD2. BV2 cells were seeded at a density of 5 × 10^5^ cells per well for 24 h and then treated with 1 μg/ml of neuromelanin for 12 h and 24 h. For comparative purposes, we also quantified the different mRNAs in response to lipopolysaccharide.

**Figure 6:**
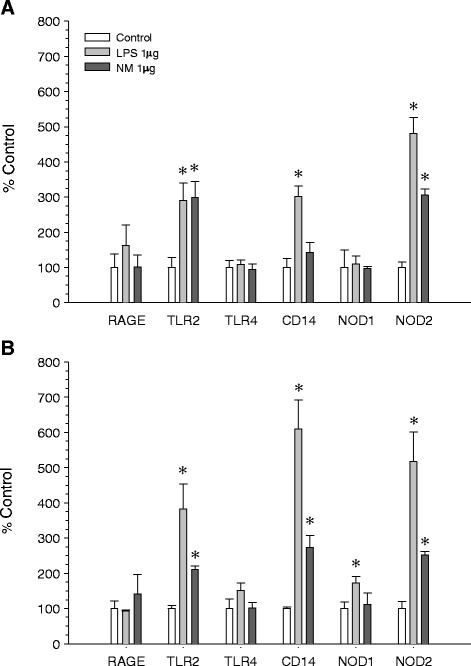
**Expression of pattern-recognition receptors in response to synthetic neuromelanin.** mRNA levels were quantified using real-time RT-PCR. BV2 cells were cultured for 24 h and then treated with 1 μg/ml of neuromelanin for 12 h and **(B)** 24 h. Results are mean ± standard deviation of three independent experiments, and are expressed as percentage of control values. Statistical analysis was made using Student’s *t* test. *, *P* < 0.01 compared with control values. LPS, lipopolysaccharide; NM, neuromelanin.

Neuromelanin induced a significant increase (*P* < 0.01) in the mRNA expression of TLR2 (298.4% of control value) and NOD2 receptors (305.8% of control value) at 12 h (Figure 6A), which then slightly decreased at 24 h (211.0% and 252.1% of control value for TLR2 and NOD2, respectively; *P* < 0.01 with respect to control value), when we observed a high induction of CD14 gene mRNA expression (272.8% of control value, *P* < 0.01; Figure 6B).

### Analysis of caspase-3 and -8 activities in response to synthetic neuromelanin

**Figure 7:**
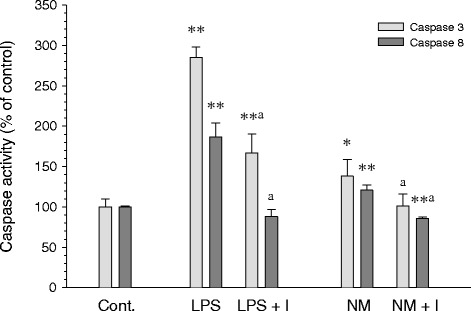
**Neuromelanin-induced caspase-3 and -8 activities.** Caspase 3 and 8 activities were assessed using the Caspase-Glo® Assay (Promega). Results are mean ± standard deviation of at least three independent experiments, and are expressed as percentage of control values. Statistical analysis: one-way ANOVA followed by the least significant difference post-hoc test for multiple comparisons. *α* = 0.05 was used. *, *P* < 0.05; **, *P* < 0.01 compared with the control group; a, compared with the same treatment without the inhibitor IETD-fmk, *P* < 0.01. Cont., control; LPS, lipopolysaccharide; LPS + I, lipopolysaccharide plus IETD-fmk; NM, neuromelanin; NM + I, neuromelanin plus IETD-fmk.

Caspases -3 and -8 are well known members of the killing caspases, but they also have non-apoptotic roles. It has been recently demonstrated that their moderate activation is involved in the activation of microglia in response to different proinflammogens. Consequently, caspase-3 and -8 activities were assessed using a Caspase-Glo® assay (Promega) in response to neuromelanin and in the presence or absence of IETD-fmk, an inhibitor of caspase-8 activity, added to the culture 24 h before neuromelanin or lipopolysaccharide (Figure 7). Neuromelanin induced a modest but still significant increase in the activity of caspase-3 (138.1% of control values, *P* < 0.05) and caspase-8 (120.9% of control value, *P* < 0.01; Figure 7). IETD-fmk reduced both lipopolysaccharide- and neuromelanin-induced caspase-3 and -8 activities.

### Neuromelanin induces release of proinflammatory factors

**Figure 8:**
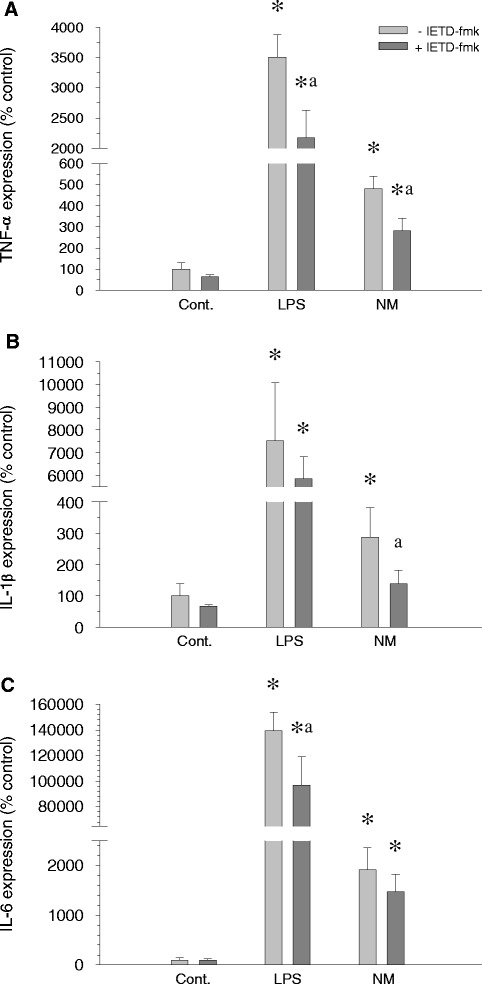
**Induction of proinflammatory factors after 6 h of exposure to neuromelanin.** mRNA expression was quantified by real-time RT-PCR 6 hours after the start of the experiments. **(A)** TNF-α. **(B)** IL-1β. **(C)** IL-6. Results are mean ± standard deviation of three independent experiments, and are expressed as percentage of control values. Statistical analysis: one-way ANOVA followed by the least significant difference post-hoc test for multiple comparisons. *α* = 0.05 was used. *, compared with control value, *P* < 0.01; a, compared with the same treatment without the inhibitor IETD-fmk, *P* < 0.01. LPS, lipopolysaccharide; NM, neuromelanin.

**Figure 9:**
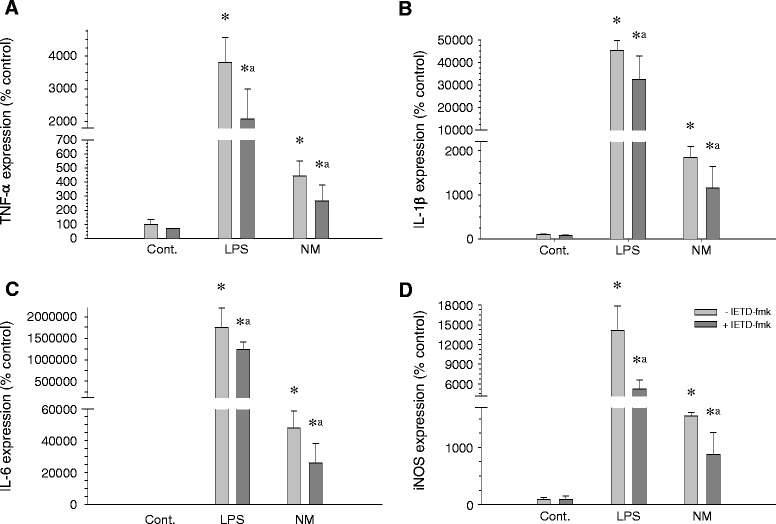
**Induction of proinflammatory factors after 24 h of exposure to neuromelanin.** mRNA expression was quantified by real-time RT-PCR 24 h after the start of the experiments. **(A)** TNF-α. **(B)** IL-1β. **(C)** IL-6. **(D)** iNOS. Results are mean ± standard deviation of three independent experiments, and are expressed as percentage of control values. Statistical analysis: one-way ANOVA followed by the least significant difference post-hoc test for multiple comparisons. *α* = 0.05 was used. *, compared with control value, *P* < 0.01; a, compared with the same treatment without the inhibitor IETD-fmk, *P* < 0.01. LPS, lipopolysaccharide; NM, neuromelanin.

We used RT-PCR to examine the level of expression of different cytokine genes in microglial cell cultures after 6 h and 24 h of treatment with neuromelanin and in the presence and absence of IETD-fmk. After stimulation of BV2 cells with neuromelanin, PCR analysis revealed an increased and time-dependent effect of neuromelanin on the mRNA levels for *TNF-α*, *IL-1β*, *IL-6*, and *iNOS* gene expression. After 6 h of exposure, an increase in *TNF-α*, *IL-1β*, and *IL-6* gene expression was evident (Figure 8); at 24 h, an increase of *iNOS* expression was also evident (Figure 9). When the treatment with neuromelanin was preceded by a 24 h treatment with IETD-fmk, a reduction of gene expression was observed, demonstrating a direct involvement of caspase-8 in proinflammatory factor induction in the presence of proinflammatory stimuli (Figures 8 and 9).

### ROS generation in response to synthetic neuromelanin

**Figure 10:**
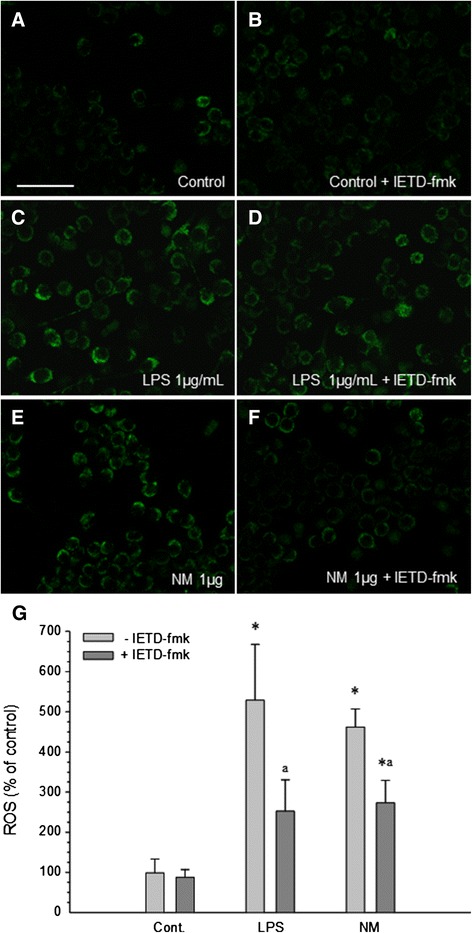
**Production of ROS induced by neuromelanin.** The fluorogenic probe CellROX® becomes fluorescent upon oxidation by ROS. **(A)** Control. **(B)** Control + IETD-fmk. **(C)** Neuromelanin 1 μg. **(D)** Neuromelanin 1 μg + IETD-fmk. **(E)** Lipopolysaccharide 1 μg. **(F)** Lipopolysaccharide 1 μg + IETD-fmk. **(G)** Quantification of ROS production. Results are mean ± standard deviation of three independent experiments, and are expressed as a percentage of control values. Statistical analysis: one-way ANOVA followed by the least significant difference post-hoc test for multiple comparisons. *α* = 0.05 was used. *, compared with control value, *P* < 0.01; a, compared with the same treatment without the inhibitor IETD-fmk, *P* < 0.01. Scale bar, 50 μm. Cont., control; LPS, lipopolysaccharide; NM, neuromelanin; ROS, reactive oxygen species.

Microglia are known to produce ROS upon activation. Intracellular ROS generation was observed in BV2 cells after a 24 h exposure to neuromelanin (Figure 10). CellROX®, a fluorogenic probe that exhibits fluorescence upon oxidation by ROS, was used. The intensity of the fluorescent signal, reflecting the amount of ROS generation, showed significant increases in reactive oxygen species in cells treated with neuromelanin and lipopolysaccharide (Figure 10). Significantly less ROS generation was observed when BV2 cells were first pre-treated for 24 h with IETD-fmk, a selective caspase-8 inhibitor, and then exposed to lipopolysaccharide or neuromelanin.

### IETD-fmk inhibits the activation of microglia induced by neuromelanin

**Figure 11:**
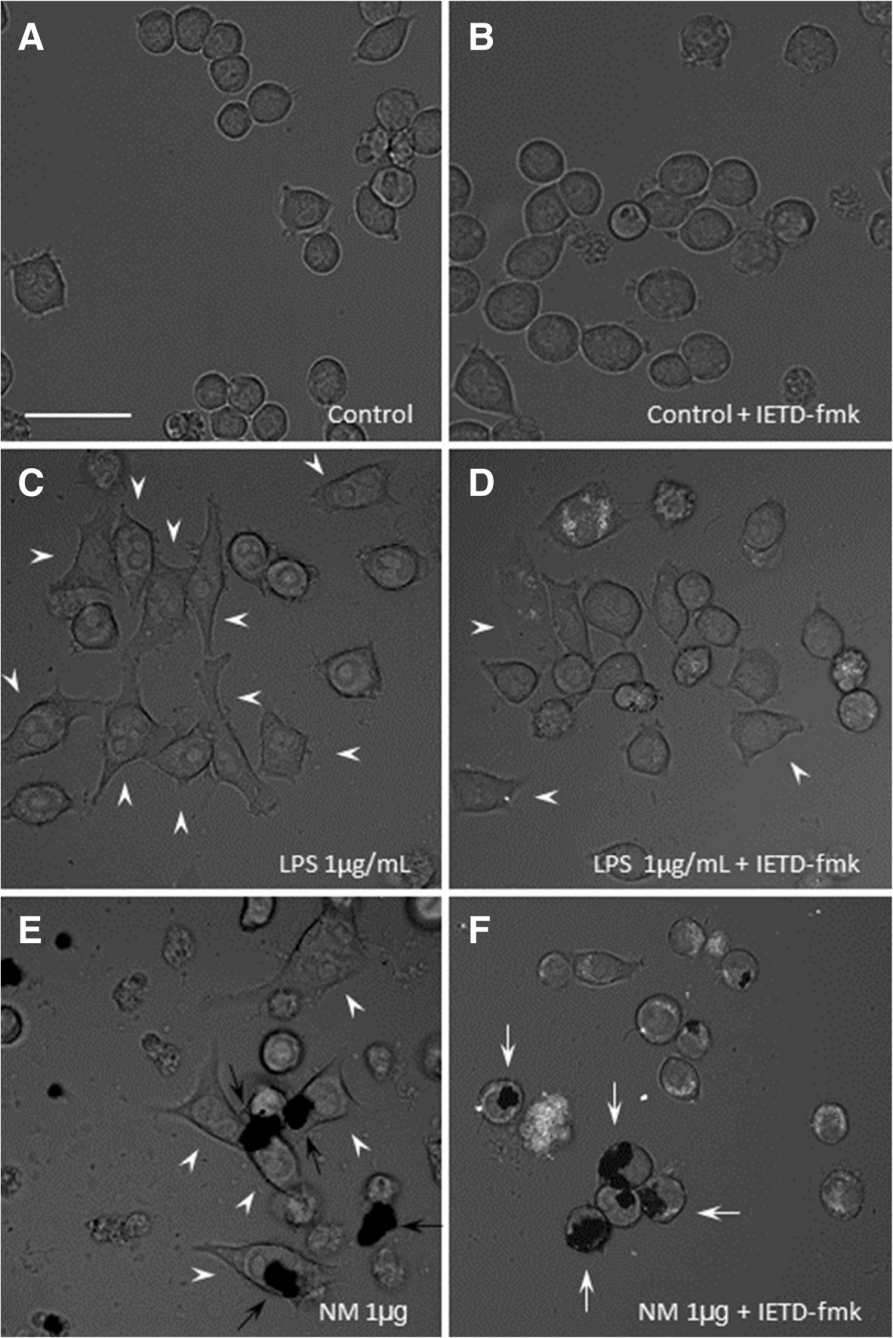
**IETD-fmk inhibits microglial activation induced by neuromelanin.** Phase contrast micrograph of BV2 cultures under different treatments. **(A)** Control cells, showing the round morphology characterizing resting microglial cells in culture. **(B)** The caspase-8 inhibitor IETD-fmk showed no effect on resting cells. **(C)** Lipopolysaccharide, used as a positive control, induced clear changes in the cells morphology (white arrowheads), which lost their resting round shape. **(D)** IETD-fmk reduced the activation induced by lipopolysaccharide in the BV2 cells. **(E)** Neuromelanin (black deposits marked by black arrows) induced a clear activation of BV2 cells, in a similar way to the proinflammogen lipopolysaccharide. **(F)** Inhibition of caspase-8 prevented the activation of BV2 cells induced by neuromelanin. Round resting cells in close contact with neuromelanin can be seen (white arrows). Scale bar, 50 μm. LPS, lipopolysaccharide; NM, neuromelanin.

A clear chemoattraction and morphological change typical of activation is evident in BV2 cells in response to neuromelanin treatment. Caspase-8 inhibition by IETD-fmk clearly prevented neuromelanin-induced morphological changes associated with microglia activation (rounded to fusiform-shaped cells) (Figure 11). To achieve this, cells were exposed to IETD-fmk for 24 h and then to neuromelanin for an additional 24 h. The effect of lipopolysaccharide was also tested, for comparative purposes.

## Discussion

We have previously reported an unexpected non-apoptotic role of caspases in the control of microglia activation and associated neurotoxicity [7]. We found that stimulation of microglia with various inflammogens activates caspase-8 and caspase-3/7 in the absence of cell death *in vitro* and *in vivo*. Knockdown or chemical inhibition of either one, or both, of these caspases hindered microglia activation and, importantly, reduced neurotoxicity. We also observed that these caspases are activated in microglia in the ventral mesencephalon in subjects with Parkinson’s disease. The question that arises deals with the potential endogenous inflammogens capable of activating microglia by a caspase-dependent mechanism.

Since the first indications for an active immune system in the ventral mesencephalon of patients with Parkinson’s disease (the presence of cytotoxic T lymphocytes and reactive microglia) were reported [23,24], numerous studies have suggested a prominent role of brain inflammation in the etiopathology of Parkinson’s disease [25–27]. Studies on brains from human beings [28] who had self-administered MPTP, and monkeys [29] receiving the same toxin, besides showing a striking microglial activation, showed a significant accumulation of extraneuronal neuromelanin, a clear indication that neuromelanin might have an active role in activating microglia in the parkinsonian brain. Supporting this, neuromelanin has been demonstrated surrounded by, or within, activated microglia in the parkinsonian brain post-mortem [30], and the number of activated microglia in the aging substantia nigra is significantly and positively correlated with the amount of extracellular neuromelanin. [31]. Furthermore, neuromelanin has been shown to stimulate neuroinflammation and cell loss when injected into the substantia nigra of the rat [18]. Interestingly, Double *et al.* [32] have demonstrated that sera from subjects with clinical Parkinson’s disease display a significantly enhanced IgG response to melanins derived from catecholamines. This finding is suggestive of a specific humoral immune response stimulated by the release of neuromelanin from dying dopaminergic neurons into the extraneuronal space and its subsequent removal from the brain by microglia [33–35]. The main precursor to neuromelanin is dopamine and cysteine appears to be partially incorporated [22]. With these precedents, we used a synthetic melanin from dopamine and cysteine. Because of the limited availability of neuromelanin [36], it is very important to develop a good model for producing synthetic neuromelanin that allows systematic studies aimed at elucidating, for instance, molecular pathways triggering brain inflammation. With this consideration, Zecca and colleagues developed a method for producing neuromelanin from autoxidation of dopamine in the presence of cysteine [37].

We first tested the ability of synthetic neuromelanin to activate microglia *in vivo*. Thus, we injected neuromelanin in the ventral mesencephalon and analyzed the morphological features of Iba1-immunolabeled microglia. Even though we found morphological changes typically associated with microglia activation, we also performed double Iba1-CD16/32 immunohistochemical analysis. CD16/32, when expressed in microglia, is a phenotypic marker of M1 polarization [38,39]. We found a clear upregulation of CD16/32 in Iba1-immunolabeled microglia, consistent with the microglia activating effect of synthetic neuromelanin. Next, we set up a cell culture system to test the ability of synthetic neuromelanin to activate microglia and the potential role of caspase-8 in neuromelanin-induced microglia activation. For that, we took advantage of BV2 cells, which we have successfully used in the context of caspase-8/3/7-dependent microglia activation in response to classical inflammogens including lipopolysaccharide and IFNγ [7]. Since caspases are critically involved in apoptotic cell death, we first analyzed the effect of increasing doses of neuromelanin in cell survival and apoptosis. Doses of up to 50 μg neuromelanin did not compromise BV2 cell survival. The neuromelanin dose selected for most of these studies (1 μg) failed to induce apoptosis as demonstrated by annexin V and propidium iodide fluorescence-activated cell sorting. We next tested the ability of 1 μg neuromelanin to induce microglia activation. To achieve this, we demonstrated (i) a positive chemotaxis assay, (ii) an increase of nitrite levels, a stable breakdown product of nitric oxide [40], and (iii) significant upregulation of cytokine mRNAs, including IL-1β, TNF-α, IL-6, and increased iNOS expression. Upon activation, expression of pattern-recognition receptors is increased in microglia [15]. More importantly, expression levels for TLR2, TLR5, and CD14 are increased in Parkinson’s disease [15,41,42]. Consequently, we extended our study to key pattern-recognition receptors, including TLR2, TLR4, CD14, RAGE, NOD1, and NOD2. For comparative purposes, we also studied the effect of lipopolysaccharide. Interestingly, both neuromelanin and lipopolysaccharide rendered quite similar results, including upregulation of TLR2, CD14, and NOD2 but not TLR4. Taken together, our results demonstrate that synthetic neuromelanin is a potent proinflammogen *in vivo* and *in vitro*, even mimicking key molecular events associated with a classical inflammatory response, like that induced by lipopolysaccharide. However, in contrast with lipopolysaccharide, neuromelanin should be considered a clear inducer of sterile inflammation (that occurring in the absence of any microorganism), which is supposed to play critical roles in neurodegenerative diseases.

It is becoming clear that the non-apoptotic roles of killing caspases are associated with certain cellular compartments (that is, nuclear versus cytoplasmic), along with a restricted timing and intensity of signal pathway activation [7,15,43]. In agreement, we demonstrated a moderated yet significant increase of caspase-8 and caspase-3 in BV2 microglia in response to classical proinflammogens, including lipopolysaccharide (TLR4 agonist), lipoteichoic acid (TLR2 agonist), PamC3sk4 (synthetic lipopeptide TLR1/2 agonist), and IFNγ, in contrast with a death stimulus induced by staurosporine, which led to significantly greater caspase activity [7]. Consequently, we measured DEVDase (caspase-3/7) and IETDase (caspase-8) activities in response to neuromelanin. Our data demonstrate that, concomitant with microglia activation, both activities were moderately yet significant increased after neuromelanin treatment. As stated before, even the highest neuromelanin doses tested failed to induce significant cell death, a prerequisite to exclude the neuromelanin-induced IETDase and DEVDase activities in triggering cell death. Consequently, we next tested the ability of IETD-fmk, a specific caspase-8 inhibitor, to counteract neuromelanin-induced proinflammatory microglia activation. Oxidative stress generated by reactive microglia is supposed to be the most critical factor in inducing the death of neuronal populations [44], and it is highly relevant for the pathogenesis of Parkinson’s disease, as nigral dopamine neurons are highly vulnerable to oxidative stress [8]. Consequently, we used a fluorogenic probe to measure cellular oxidative stress in response to neuromelanin in the presence and absence of IETD-fmk, a specific caspase-8 inhibitor. From these experiments, it was clear that (i) BV2 cells showed a clear positive chemotaxis and moved towards areas with insoluble neuromelanin, (ii) neuromelanin induced morphological changes typical of microglia activation; (iii) neuromelanin-treated cells strongly increased oxidative stress, and (iv) caspase-8 inhibition prevented typical morphological features of microglia activation and associated oxidative stress. In keeping with this view, activated microglia upregulates different enzymes involved in the inflammatory processes mediated by oxidative stress, including iNOS, NADPH oxidase, COX-2, and myeloperoxidase [15]. Moreover, stimulated microglia upregulate a wide variety of proinflammatory mediators including cytokines. All these events related to microglia activation potentially contribute to neuronal dysfunction and death. Consequently, and to further confirm that caspase-8 inhibition prevents neuromelanin-induced microglia activation, we measured main proinflammatory cytokines including IL-1β, IL-6, TNF-α, and expression of iNOS. Our results demonstrated that caspase-8 inhibition partially but significantly prevented neuromelanin-induced upregulation of the mentioned cytokines and iNOS. Taken together, our results demonstrate a caspase-dependent activation of microglia in response to synthetic neuromelanin.

## Conclusions

We demonstrated that synthetic neuromelanin is an efficient proinflammogen *in vivo* and *in vitro*. Neuromelanin also exhibited a very significant chemotactic response to BV2 microglial cells. Analysis of IETDase and DEVDase activities in BV2 cells demonstrated a modest yet significant increases of caspase-8 and caspase-3 activities in response to neuromelanin treatment. A dose-response study demonstrated the ability of neuromelanin to activate microglia in the absence of cell death. Consequently, the neuromelanin-induced activation of caspase-8 and caspase-3/7 are suggestive of non-apoptotic roles of these killing caspases. Supporting this, caspase-8 inhibition prevented typical features of microglial activation, including morphological changes, a high rate of oxidative stress and expression of key proinflammatory cytokines and iNOS. Neuromelanin released by damaged dopaminergic neurons is suspected to play a major role in brain inflammation associated with Parkinson’s disease. Elucidating molecular mechanisms associated with neuromelanin-induced microglia activation are crucial for establishment of future pharmacological strategies aimed at preventing microglia neurotoxicity. Our data demonstrate a key role of caspase-8 in microglia activation induced by neuromelanin.

## Abbreviations

ANOVA: analysis of variance
Ct: cycle time
DEVDase: D(OMe)E(OMe)VD(OMe)-ase
DMEM: Dulbecco’s modified Eagle’s medium
EDTA: ethylenediaminetetraacetic acid
FBS: fetal bovine serum
GAPDH: glyceraldehyde 3-phosphate dehydrogenase
IETD: Z-Ile-Glu(OMe)-Thr-Asp(OMe)-fluoromethylketone
IFNγ: interferon γ
IgG: immunoglobulin G
MPTP: 1-methyl-4-phenyl-1,2,3,6-tetrahydropyridine
MTT: 3-(4,5-dimethylthiazol-2-yl)-2,5-diphenyl tetrazolium
NOD: nucleotide-binding oligomerization domain-containing protein
PBS: phosphate buffered saline
PCR: polymerase chain reaction
qPCR: quantitative polymerase chain reaction
RAGE: receptor for advanced glycation end products
ROS: reactive oxygen species
TLR: toll-like receptor
